# Retraction: COVID-19 knowledge, attitude, and preventive practices among government and private bank workers in Ethiopia

**DOI:** 10.3389/fpubh.2026.1794532

**Published:** 2026-01-29

**Authors:** Frontiers Editorial Office

The journal retracts the October 30, 2023, article cited above.

Following publication, concerns were raised about the potential publication of duplicate content in the article cited above in Frontiers in Public Health and the following article published in PLOS One: Hassen S, Adane M (2021) Facemask-wearing behavior to prevent COVID-19 and associated factors among public and private bank workers in Ethiopia. PLOS ONE 16(12):e0259659. doi: 10.1371/journal.pone.0259659.

An investigation was conducted in accordance with Frontiers' policies, whereby it was confirmed that there is a substantial overlap and minimal new information compared to the previously published work in PLOS ONE. Therefore, following the Committee on Publication Ethics guidelines, this article, published more recently, is deemed to be redundant and as such is retracted.

This retraction was approved by the Chief Editors of Frontiers in Public Health and the Chief Executive Editor of Frontiers. The authors have not responded to correspondence regarding this retraction.

